# Long-term prognostic utility of low-density lipoprotein (LDL) triglyceride in real-world patients with coronary artery disease and diabetes or prediabetes

**DOI:** 10.1186/s12933-020-01125-1

**Published:** 2020-09-27

**Authors:** Jing-Lu Jin, Hui-Wen Zhang, Ye-Xuan Cao, Hui-Hui Liu, Qi Hua, Yan-Fang Li, Yan Zhang, Yuan-Lin Guo, Na-Qiong Wu, Cheng-Gang Zhu, Rui-Xia Xu, Ying Gao, Xiao-Lin Li, Chuan-Jue Cui, Geng Liu, Jing Sun, Qian Dong, Raul Santos, Jian-Jun Li

**Affiliations:** 1 State Key Laboratory of Cardiovascular Disease, Fu Wai Hospital, National Center for Cardiovascular Diseases, Chinese Academy of Medical Sciences, Peking Union Medical College, BeiLiShi Road 167, Beijing, 100037 China; 2 Department of Cardiology, Xuanwu Hospital, Capital Medical University, Beijing, 100053 China; 3 Department of Cardiology, Beijing Anzhen Hospital, Capital Medical University, Beijing, 100029 China; 4 Hospital Israelita Albert Einstein, Sao Paulo, Brazil; 5 Heart Institute (InCor), University of Sao Paulo Medical School Hospital, Sao Paulo, Brazil

**Keywords:** LDL-TG, Stable CAD, Diabetes, Pre-diabetes, MACEs

## Abstract

**Background:**

Recent guidelines highlighted the association between atherosclerosis and triglyceride-enriched lipoproteins in patients with impaired glucose metabolism. However, evidence from prospective studies for long-term prognostic utility of low-density lipoprotein triglyceride (LDL-TG) in real-world patients with prediabetes (Pre-DM) or diabetes mellitus (DM) and coronary artery disease (CAD) is currently not available. The aim of the present study was to evaluate the impact of LDL-TG on major adverse cardiovascular events (MACEs) in patients with stable CAD under different glucose metabolism status.

**Methods:**

A total of 4381 patients with CAD were consecutively enrolled and plasma LDL-TG level was measured by an automated homogeneous assay. They were categorized according to both status of glucose metabolism [DM, Pre-DM, normal glycaemia regulation (NGR)] and tertiles of LDL-TG. All subjects were followed up for the occurrence of MACEs.

**Results:**

During a median of 5.1 (interquartile range 3.9 to 5.9) years’ follow-up, 507 (11.6%) MACEs occurred. Cubic spline models showed a significant association between LDL-TG and MACEs in DM and Pre-DM but not in NGR. When the combined effect of elevated LDL-TG and glucose disorders was considered for risk stratification, the medium tertile of LDL-TG plus DM, and the highest tertile of LDL-TG plus Pre-DM or plus DM subgroups were associated with significantly higher risk of MACEs after adjustment of confounders including triglyceride [hazard ratios (95% confidence intervals): 1.843 (1.149–2.955), 1.828 (1.165–2.867), 2.212 (1.396–3.507), all p < 0.05]. Moreover, adding LDL-TG into the original model increased the C-statistic from 0.687 to 0.704 (∆C-statistic = 0.016, p = 0.028) and from 0.734 to 0.749 (∆C-statistic = 0.014, p = 0.002) in Pre-DM and DM, respectively.

**Conclusions:**

In this longitudinal cohort study on real-world practice, higher LDL-TG was associated with worse outcomes among Pre-DM and DM patients with stable CAD.

## Background

Genetic studies have verified that both plasma triglyceride (TG) levels or genetic variants leading to hypertriglyceridemia are independently associated with higher risk of coronary artery disease (CAD) [1, 2]. In the statin era, even among those with low density lipoprotein cholesterol (LDL-C) within recommended values, elevation in plasma TG still indicates an elevated residual risk. Hence, TG-enriched lipoproteins could be a causal risk factor for cardiovascular events [3]. However, in randomized clinical trials, inconsistent results about CAD risk reduction were reported among patients receiving TG lowering therapies [4–6]. Although TGs might not be atherogenic per se, several studies showed that TG-enriched lipoproteins were strongly related with the occurrence of atherosclerotic cardiovascular disease (ASCVD) events [7–9].

Low density lipoprotein triglyceride (LDL-TG), which represents the TG content of LDL particles, was previously reported to be associated with incident cardiovascular disease. In the Atherosclerosis Risk in Communities (ARIC) study, the prognostic value of LDL-TG trumped that of remnant cholesterol (RC) in patients without CAD at baseline [7]. As it was reported by McKeone et al., LDL with elevated TG contents formed in vitro had reduced proteolytic cleavage of apo B-100 [10]. For real-world individuals, plasma levels of LDL-TG were elevated when more TGs were transferred from very low density lipoprotein (VLDL) to LDL via cholesteryl ester transfer protein (CETP) [11]. Notably, this hypertriglyceridemia-mediated pathway was more active when individuals presented glycemic metabolism disorders [11, 12].

Diabetes mellitus (DM) is a common disease which confers a two-fold excess risk of vascular diseases after adjustment for confounders [13]. In type 2 diabetes mellitus(T2DM) patients, the macrovascular complications are due in part to hyperglycemia, but the coexistence of other disorders may result in worse prognosis [14, 15]. The morbidity rate of pre-diabetes (Pre-DM), a state of mild glucose dysregulation, is also increasing worldwide. The incidence rate of Pre-DM is almost three-fold higher than that of DM [16]. Interestingly, although Pre-DM individuals present modestly raised glucose levels, similar cardiac abnormalities of DM subjects were also observed in these patients. For example, Coopmans et al. has reported that Pre-DM and DM are similarly associated with reduction of heart rate variability [17]. Previous studies including ours have also indicated that patients with DM or Pre-DM have similar pro-atherogenic lipid profiles [12]. Compared with normal glucose regulation (NGR) individuals, lipid abnormalities, especially for hypertriglyceridemia and elevated lipoprotein (a), were more significantly associated with cardiovascular events in DM or Pre-DM patients [14, 18]. Moreover, several studies indicated that increased plasma LDL-TG level was one of the typical changes encountered in patients with impaired glucose metabolism [12]. This raised the possibility that higher LDL-TG levels may have different impacts on the prognosis of patients with NGR, Pre-DM and DM. In the current study, we aim to investigate the different relations of LDL-TG levels to major cardiovascular events (MACEs) in stable CAD patients with different glucose metabolism status under real-world clinical practice.

## Method

### Study design and participants

This study complied with the Declaration of Helsinki and was approved by the local ethical review board. Informed written consents were obtained from all participants.

Study patient disposition is described in the flowchart at Additional file 1: Figure S1. From March 2011 to December 2016, 5132 patients were admitted to three medical centers and diagnosed as angiography-proven CAD. Seven-hundred and fifty-one patients did not enter the final analysis for the following exclusion criteria: missing data, having suffered an acute coronary syndrome (ACS) or previous revascularization, decompensated heart failure, severe liver and/or renal insufficiency, thyroid dysfunction, systematic inflammatory disease, malignant disease, and chylomicronemia. Patients were followed up by telephone or presential interviews at 6 months’ intervals by experienced nurses or physicians. Medical records of those who reported MACEs were checked by experienced physicians who were blinded to the current study. The MACEs were defined as cardiovascular mortality, non-fatal myocardial infarction (MI), atherothrombotic stroke, unplanned percutaneous coronary intervention (PCI) or coronary artery bypass grafting (CABG), and hospitalization for unstable angina. Non-fatal myocardial infarction was diagnosed as positive cardiac enzymes along with typical chest pain or electrocardiogram serial changes. Stroke was diagnosed by medical history, typical symptoms, and characterized imaging.

DM was diagnosed by fasting plasma glucose (FPG) ≥ 7.0 mmol/L or the 2-h plasma glucose of the oral glucose tolerance test ≥ 11.1 mmol/L, haemoglobin A1c (HbA1c) level ≥ 6.5% or currently using anti-diabetic drugs or insulin. Pre-DM was diagnosed in participants who did not have medical history of DM but met the American Diabetes Association(ADA) criteria of Pre-DM [fasting plasma glucose 5.6 to 6.9 mmol/L, 2-h glucose ranging from 7.8 to 11.0 mmol/L, or hemoglobin A1c (HbA1c) level from 5.7 to 6.4%] [19]. Patients without DM or pre-DM were categorized as NGR. Hypertension was defined as medical history of hypertension, currently receiving antihypertensive drugs or hospital recorded systolic blood pressure (SBP) ≥ 140 mmHg and/or diastolic blood pressure (DBP) ≥ 90 mmHg for three or more consecutive times. Other clinical characters, including family history of early cardiovascular disease, prior medications, smoking, and alcohol consumption, were collected from self-reported or hospital-recorded medical history.

### Laboratory analysis

Blood samples were obtained after 12-h fasting once upon admission and were collected into EDTA-containing tubes. After centrifugation at 3000 rpm for 10 min at 4 °C, plasma was collected and stored at − 80 °C. Plasma concentrations of total cholesterol (TC), TG, LDL-C, high density lipoprotein cholesterol (HDL-C), apolipoprotein B (ApoB) were measured by automatic biochemistry analyzer (Hitachi 7150, Tokyo, Japan) in an enzymatic assay. Non-HDL-C was calculated as TC minus HDL-C. Plasma levels of LDL-TG were measured by an automated homogeneous assay (DENKA SEIKEN CO., LTD, Tokyo, Japan) [20]. The measured LDL-TG was with calibration range of 0.0–80 mg/dL. The automated homogeneous LDL-TG method used for our study was validated against the standard sequential density ultracentrifugation method [20]. The concentrations of glucose were measured by enzymatic hexokinase method. HbA1c was measured using Tosoh Automated Glycohemoglobin Analyser (HLC-723G8, Tokyo, Japan).

### Evaluation of CAD severity

Angiographic data were collected according to catheterization laboratory records. Three experienced interventional physicians evaluated the result of coronary angiography for each patient, and CAD severity was determined using the Gensini score (GS) as previously described [21].

### Statistical analysis

The values for the continuous variables and the categorical variables were presented as the mean ± standard deviation (SD), median (Q1–Q3) or number (percentage). The Kolmogorov–Smirnov test was used to test the distribution pattern. The differences of variables among groups were analyzed using Student *t* test, analysis of variance, or nonparametric test where appropriate. The Kaplan–Meier method was used to estimate the event-free survival rates among. The log-rank test was used to test the statistical significance. The hazard ratios (HRs) and 95% confidence intervals (CI) were calculated by univariate and multivariate Cox regression analyses. The association of LDL-TG concentration with MACEs occurrence was tested either as categorical (divided in low (T1), medium (T2) and high (T3) to tertiles) or continuous (per 1-SD) variables. Both Kaplan–Meier method and Cox regression analyses were performed in subgroups according to DM status and tertiles of LDL-TG levels. HRs for MACEs were also calculated for participants who were divided into 9 groups by both status of glucose metabolism and LDL-TG levels using NGR plus low LDL-TG as reference. The associations between LDL-TG concentrations and MACEs of different glucose metabolism groups were also assessed by restricted cubic spline models. The concordance index(C-statistic) was calculated to test the model efficiency. The Cox analyses were also performed in DM patients to compare the HRs between those with different glucose control status and LDL-TG levels with good glucose control (HbA1c < 7%) plus low LDL-TG subgroup. The adjustments in multivariate models included age, sex, BMI, smoking, hypertension, family history of early CAD, Gensini score, LVEF, LDL-C HDL-C, TG and baseline use of statins. A p-value < 0.05 was considered statistically significant. The statistical analyses were performed with SPSS version 21.0 software (SPSS Inc., Chicago, IL, USA) and R language version 3.5.2 (Feather Spray).

## Results

### Baseline characteristics

Patients were divided into three groups according to different glucose metabolism status (NGR: n = 926, Pre-DM: n = 1789, DM n = 1666, Table 1). Older and female patients were more likely to have Pre-DM and DM (p < 0.001 and p = 0.003, respectively). The plasma levels of glucose, HbA1c, TG, LDL-TG and proportion of hypertension increased while the levels of HDL-C decreased according to different glucose metabolism status (all p < 0.001). Patients with Pre-DM had higher TC, LDL-C and non-HDL-C than those with NGR (p < 0.05). DM but not Pre-DM group showed higher body mass index (BMI) and GS but lower left ventricle ejection fraction (LVEF) and lower rate of early family history of CAD than NGR group. Non-significant differences were found regarding ApoB levels, alcohol consumption, smoking and use of medications among NGR, Pre-DM and DM subgroups (p > 0.05).

**Table 1:** Baseline clinical and laboratory characteristics according to glucose metabolism status

Variables	Total n = 4381	NGR n = 926	Pre-DM n = 1789	DM n = 1666	p value
Clinical characteristics					
Age, years	58.2 ± 9.8	55.5 ± 9.9	58.5 ± 9.5	59.3 ± 9.8	< 0.001
Male sex, n (%)	3120 (71.2)	700 (75.6)	1263 (70.6)	1157 (69.4)	0.003
BMI (kg/m^2^)	25.9 ± 3.1	25.5 ± 3.1	25.7 ± 3.1	26.4 ± 3.1	< 0.001
Hypertension, n (%)	2857 (65.2)	536 (57.9)	1141 (63.8)	1180 (70.8)	< 0.001
Family history of early CAD, n (%)	610 (13.9)	150 (16.2)	246 (13.8)	214 (12.8)	0.059
Current smoker, n (%)	2373 (54.2)	511 (55.2)	962 (53.8)	900 (54.0)	0.774
Alcohol consumption, n (%)	1400 (32.0)	311 (33.5)	576 (32.2)	513 (30.8)	0.330
Laboratory findings					
Glucose (mmol/L)	6.2 ± 2.0	4.9 ± 0.6	5.3 ± 0.7	7.8 ± 2.4	< 0.001
HbA1c (%)	6.5 ± 1.2	5.4 ± 0.2	6.0 ± 0.2	7.7 ± 1.2	< 0.001
Creatinine (μmol/L)	77.6 ± 15.7	78.3 ± 15.4	77.1 ± 15.1	77.8 ± 16.6	0.149
TC (mg/dL)	159.88 ± 41.62	157.93 ± 41.23	161.82 ± 42.79	158.71 ± 41.23	0.040
HDL-C (mg/dL)	40.85 ± 11.28	42.01 ± 12.45	43.71 ± 11.28	39.68 ± 10.50	< 0.001
LDL-C (mg/dL)	97.25 ± 36.57	95.31 ± 36.18	98.81 ± 37.34	96.47 ± 35.40	0.036
Non-HDL-C (mg/dL)	119.03 ± 40.07	115.92 ± 40.07	120.20 ± 40.56	119.03 ± 39.68	0.020
ApoB (g/L)	0.88 (0.71–1.08)	0.86 (0.70–1.07)	0.88 (0.71–1.09)	0.88 (0.72–1.08)	0.405
TG (mmol/L)	1.49 (1.10–2.11)	1.39 (1.02–1.98)	1.52 (1.12–2.10)	1.53 (1.15–2.19)	< 0.001
LDL-TG (mg/dL)	17.93 ± 6.53	16.77 ± 6.69	17.83 ± 6.45	18.67 ± 6.44	< 0.001
LVEF (%)	64.4 ± 7.2	65.0 ± 6.5	64.9 ± 6.8	63.5 ± 7.9	< 0.001
GS	22 (10–38)	20 (11–34)	20 (10–36)	24 (10–42)	0.002
Medications					
Statins, n (%)	3237 (73.9)	675 (72.9)	1310 (73.2)	1252 (75.1)	0.324
Statins at follow-up, n (%)	4250 (97.0)	892 (96.3)	1744 (97.5)	1614 (96.9)	0.226
Aspirin, n (%)	2684 (61.3)	554 (59.8)	1083 (60.5)	1037 (62.2)	0.411
ACEIs/ARBs, n (%)	1266 (28.9)	261 (28.2)	517 (28.9)	488 (29.3)	0.838
β-blockers, n (%)	2394 (54.6)	495 (53.5)	961 (53.7)	938 (56.3)	0.224
Antidiabetes drugs					
OADs, n (%)	1020 (23.3)	–	–	1020 (61.2)	–
Insulin, n (%)	561 (12.8)	–	–	561 (33.7)	–

Data were expressed as mean ± SD, median with 25th and 75th percentiles or n (%). ACEIs, ACE inhibitors; ARBs, angiotensin receptor blockers; OADs, oral antidiabetes drugs

### Glucose metabolism status, lipid parameters and coronary severity

The coronary severity was assessed by GS in different subgroups according to glucose metabolism status and LDL-TG levels. As shown in Additional file 1: Figure S2A, DM group had significantly higher GS than NGR group (p < 0.001). However, no significant difference in GS was observed between pre-DM and NGR groups (P > 0.05). In the meanwhile, high (T3) but not medium (T2) LDL-TG subgroups had higher GS than low LDL-TG subgroup (T1, Additional file 1: Figure S2B).

In both univariate and multivariate linear regression analyses (Additional file 1: Table S1), the HDL-C levels were negatively correlated with GS (p < 0.001, respectively). However, no relationship between plasma TG levels and coronary severity was found. Other lipid parameters, including TG, LDL-C, HDL-C, non-HDL-C, ApoB, and LDL-TG were positively correlated with the severity of CAD (all p < 0.05).

### Predictive value of LDL-TG and other lipid parameters for cardiovascular outcomes

Overall, during a median follow-up of 5.1 (interquartile range 3.9 to 5.9) years, 507 MACEs occurred, including 75 cardiovascular deaths (14.8%), 52 nonfatal MIs (10.3%), 110 nonfatal strokes (21.7%), 168 unplanned revascularizations (33.1%) and 102 (20.1%) unstable angina hospitalizations. The incidence rate of MACEs was 25.1 (95% CI 23.2–27.3) per 1000 person years.

As shown in Additional file 1: Table S2, for the total population, 1-SD increment of LDL-TG was associated with 26.8% and 32.8% higher risk of outcomes in univariate and multivariate models respectively (HR per 1-SD increment: 1.268, 95% CI 1.174–1.370 and adjusted HR per 1-SD increment: 1.328, 95% CI 1.202–1.467). Compared with other lipid parameters including TG, LDL-C, HDL-C, non-HDL-C, and ApoB, the prognostic value of LDL-TG was more significant (Table 2).

**Table 2:** Univariate and multivariate Cox proportional hazards regression analysis of the lipid parameters with MACEs

Variables	Per-SD	Univariate Cox regression		Multivariate Cox regression	
		HR(95% CI)	P	HR(95% CI)	P
TG	1.02 mg/dL	1.087 (1.002–1.180)	*0.046*	1.020 (0.930–1.119)	0.676
LDL-C	36.57 mg/dL	1.116 (1.027–1.212)	*0.009*	1.091 (1.002–1.189)	*0.046*
HDL-C	40.85 mg/dL	0.976 (0.893–1.067)	*0.599*	0.952 (0.865–1.048)	0.316
Non-HDL-C	40.07 mg/dL	1.108 (1.020–1.204)	*0.016*	1.096 (1.005–1.195)	*0.038*
ApoB	0.29 g/L	1.089 (1.006–1.179)	*0.034*	1.069 (0.987–1.159)	0.102
LDL-TG	6.53 mg/dL	1.268 (1.174–1.370)	< *0.001*	1.310 (1.192–1.439)	< *0.001*

Italics values indicate statistically significant

Multivariate adjustments include age, sex, body mass index, smoking, hypertension, diabetes mellitus, family history of early coronary artery disease, Gensini score, left ventricular ejection fraction, and baseline statin use

### LDL-TG, different glucose metabolism status and occurrence of MACEs

**Fig. 1:**
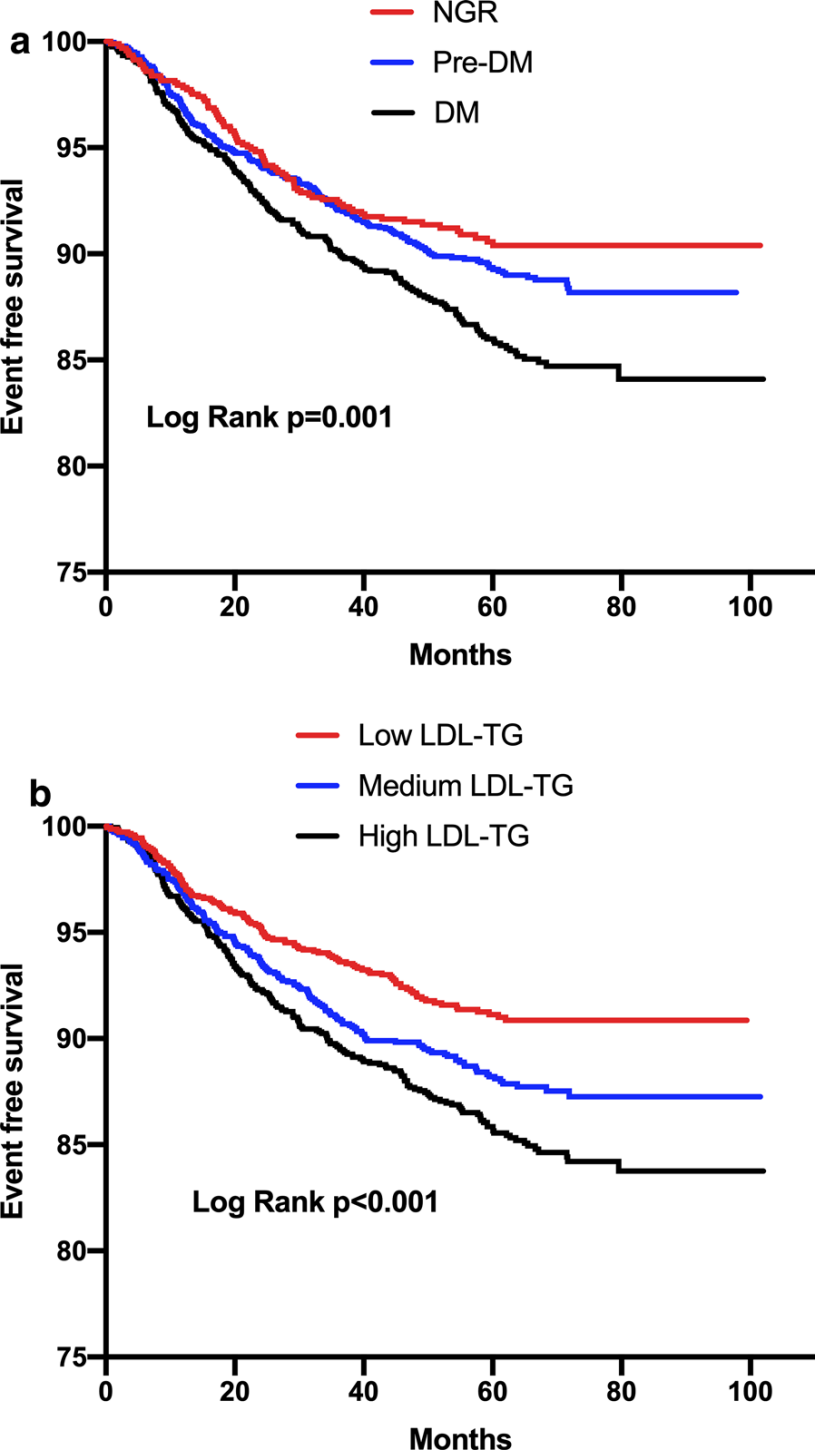
Kaplan-Meier analysis according to (**a**). different glucose metabolism status (NGR- normal glucose regulation; Pre-DM- prediabetes; DM-diabetes mellitus); **b** different LDL-TG levels (low T1; medium T2 and high T3)

Kaplan–Meier analysis (Fig. 1a) showed that DM subjects had significantly lower event-free survival rates than NGR (P < 0.05), while there were non-significant differences between those of pre-DM and NGR. Both medium (T2) and high LDL-TG subgroups (T3) had higher event rates than low LDL-TG subgroup (T1, Fig. 1b).

**Fig. 2:**
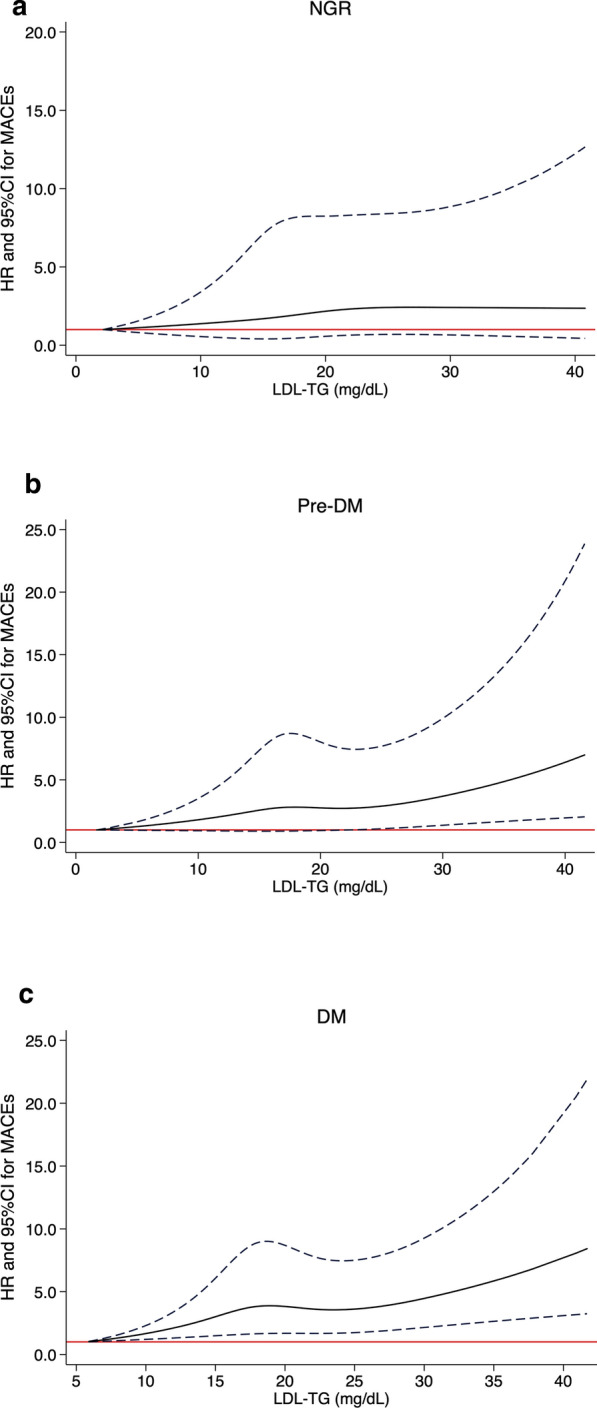
Restricted cubic spline plot of the association between LDL-TG levels and the risk of MACEs in **a** NGR **b** Pre-DM and **c** DM groups. Solid lines: hazard ratio (HR); dashed lines: 95% confidence limits (CL); red lines: reference

**Fig. 3:**
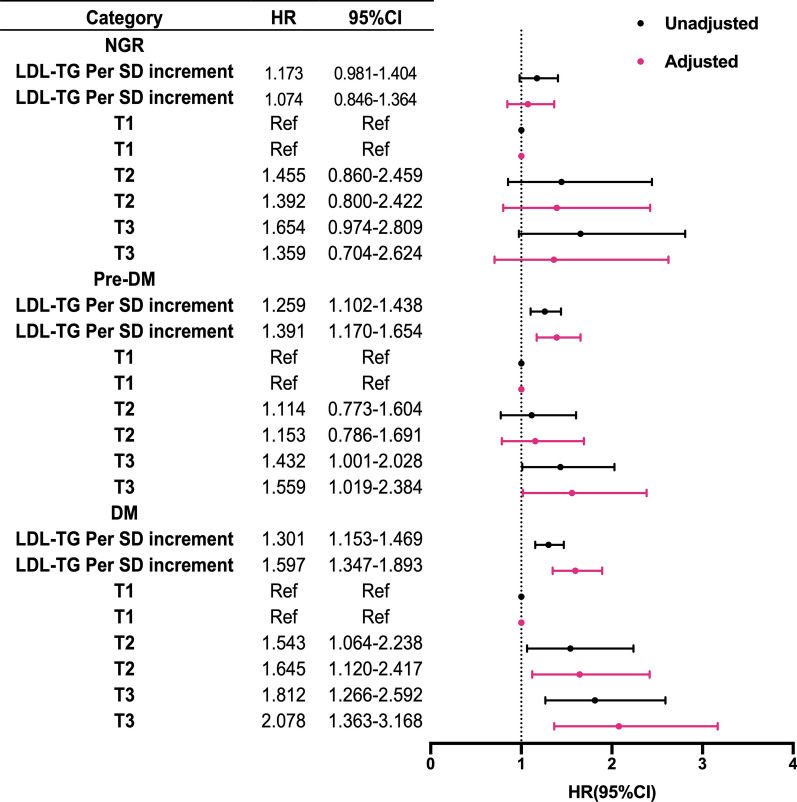
Predictive value of continuous and categorical LDL-TG in different glucose metabolism status. Glucose metabolism status (NGR- normal glucose regulation; Pre-DM- pre diabetes; DM-diabetes mellitus); LDL-TG levels (low T1; medium T2 and high T3); adjusted variables include age, sex, body mass index, smoking, hypertension, family history of early coronary artery disease, Gensini score, left ventricular ejection fraction, LDL-cholesterol, HDL-cholesterol, triglyceride and baseline statins

Restricted cubic spline models showed that no association between LDL-TG and MACEs existed in NGR group (Fig. 2a). On the other hand, pre-DM and DM groups presented increasing associations between LDL-TG and MACEs while such association was stronger in the DM group (Fig. 2b, c). As presented in Fig. 3, LDL-TG (per SD increment) as a continuous variable was positively associated with MACEs occurrence in patients with Pre-DM and DM (adjusted HR 1.391, 95% CI 1.170–1.654 and HR: 1.597, 95% CI 1.347–1.893, respectively, all p < 0.05) but not in those with NGR (HR 1.074, 95% CI 0.846–1.364 p > 0.05). When LDL-TG was analyzed according to tertiles (low, medium and high LDL-TG for T1 to T3), high LDL-TG subgroups presented 1.559-fold and 2.078-fold higher risk of MACEs than low LDL-TG subgroups in patients with Pre-DM and DM. Medium LDL-TG (T2) had higher risk of MACEs in DM (adjusted HR: 1.645, 95% CI 1.120–2.417, p < 0.05) but not in Pre-DM (adjusted HR: 1.153, 95% CI 0.786–1.691, p > 0.05). Both high and medium LDL-TG subgroups did not present higher events risk in NGR (all p > 0.05).

As presented in Additional file 1: Table S3, univariate Cox regression models showed that patients with DM had 1.529-fold higher risk of MACEs while pre-DM group did not present increment in MACEs. Additional adjustment for age and sex or traditional confounders did not change the association. However, when both glucose metabolism and LDL-TG status were incorporated as stratifying factors, multivariate adjusted Cox regression analyses indicated that patients in DM plus medium (T2) LDL-TG, pre-DM plus high LDL-TG (T3) and DM plus high LDL-TG (T3) had higher risk of MACEs than individuals with NGR plus low LDL-TG respectively [HR(95% CI) 1.843 (1.149–2.955), 1.828 (1.165–2.867), 2.212 (1.396–3.507), all p < 0.05, Table 3].

**Table 3:** LDL-TG levels in relation to cardiovascular events in patients with different glucose metabolism status

LDL-TG	Events/subjects	HR (95% CI)	
	507/4381	Crude model	Adjusted model
NGR			
Low LDL-TG (T1)	26/378	Ref	Ref
Medium LDL-TG (T2)	30/296	1.443 (0.853–2.440)	1.491 (0.877–2.535)
High LDL-TG (T3)	29/252	1.654 (0.974–2.808)	1.663 (0.958–2.866)
Pre-DM			
Low LDL-TG (T1)	54/603	1.278 (0.800–2.040)	1.155 (0.723–1.846)
Medium LDL-TG (T2)	62/608	1.443 (0.913–2.282)	1.368 (0.859–2.179)
High LDL-TG (T3)	77/578	1.873 (1.201–2.923)*	1.843 (1.149–2.955)*
DM			
Low LDL-TG (T1)	42/460	1.343 (0.824–2.191)	1.116 (0.682–1.825)
Medium LDL-TG (T2)	82/581	2.084 (1.341–3.240)*	1.828 (1.165–2.867)*
High LDL-TG (T3)	105/625	2.457 (1.600–3.775)*	2.212 (1.396–3.507)*

Model adjusted for age, sex, body mass index, smoking, hypertension, diabetes mellitus, family history of early coronary artery disease, Gensini score, left ventricular ejection fraction, LDL-cholesterol, HDL-cholesterol, triglycerides and baseline use of statins; *for p < 0.05

Including LDL-TG to the risk model of traditional CAD risk factors improved the model efficiency by 0.017[∆C-statistic and 95% CI 0.016 (0.004–0.033), C-statistic and 95% CI 0.704 (0.664–0.742), p = 0.028, Additional file 1: Table S4] and 0.014[∆C-statistic and 95% CI 0.014 (0.006–0.024), C-statistic and 95% CI 0.749 (0.715–0.783), p = 0.002] in Pre-DM and DM, respectively.

### LDL-TG and MACEs occurrence according to DM control status

**Fig. 4:**
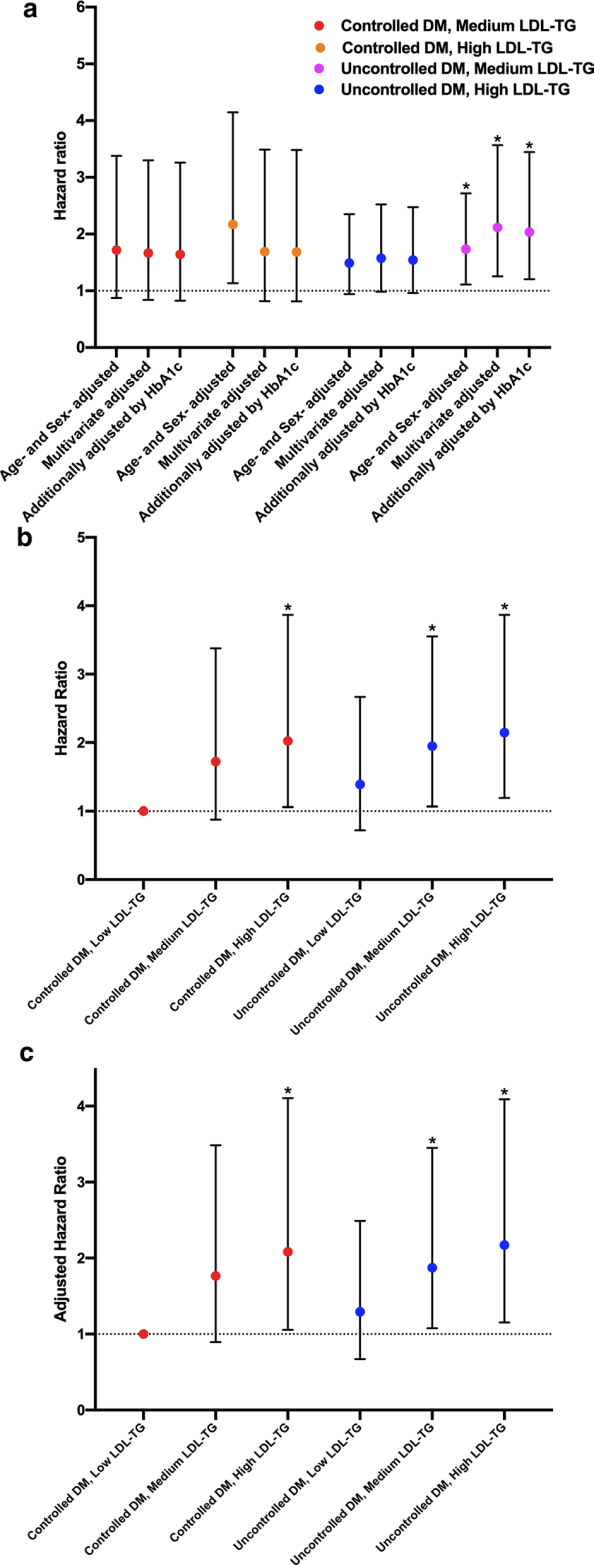
**a** Adjusted HR for medium and high LDL-TG in controlled and uncontrolled DM (**b**) unadjusted and (**c**) adjusted HR according to both LDL-TG (low T1, medium T2 and high T3) and DM controlled status (well controlled DM plus low LDL-TG as reference). Multivariate adjusted model included age, sex, body mass index, smoking, hypertension, family history of coronary artery disease, Gensini score, left ventricular ejection fraction, LDL-cholesterol, HDL-cholesterol, triglycerides, baseline statin use, and antidiabetic drugs. * for p < 0.05

In the subgroup of controlled DM (defined as HbA1c < 7% n = 572, 34.3%), both medium (T2) and high LDL-TG (T3) had no association with MACEs after adjustment for other risk factors. Meanwhile, among patients with uncontrolled DM (n = 1094, 65.7%), high LDL-TG (T3) but not medium LDL-TG (T2) was associated with higher risk of MACEs occurrence in fully adjusted models (High LDL-TG: adjusted HR: 1.932, 95% CI 1.150–3.245, p < 0.05, Fig. 4a). When DM patients were categorized into 6 groups according to both levels of HbA1c (7% as cut-off) and LDL-TG levels (Fig. 4b, c), those in controlled DM plus high LDL-TG, uncontrolled DM plus medium LDL-TG, and uncontrolled DM plus high LDL-TG had 2.082-fold (95% CI 1.055–4.106, p < 0.05), 1.874-fold (95% CI 1.077–3.451, p < 0.05) and 2.171-fold (95% CI 1.152–4.090, p < 0.05) higher risk of MACEs in multivariate adjusted models.

## Discussion

Previous study reported that plasma LDL-TG level was predictive of incident CAD in community-based populations [7]. In patient with DM, the malignant nature of diabetic dyslipidemia may be easily revealed by TGs in lipoprotein classes. In this real-world study, it was demonstrated for the first time that LDL-TG could independently predict the occurrence of MACEs in stable CAD patients with Pre-DM and DM but not in those with NGR. The results persisted after adjustment for robust risk markers including TG,LDL-C, HDL-C, coronary severity and use of lipid lowering therapies.

Hypertriglyceridemia is a common form of dyslipidemia worldwide. Approximately 30% of adult population in Western societies have elevated TG (> 1.7 mmol/L) [22]. In China, the prevalence of hypertriglyceridemia also increased from 5.7% to 15% from 2002 to 2015 [23]. Although the association between TG and CAD risk was supported by solid evidence from genome-wide analyses and Mendelian randomization studies, several TG lowering therapies had failed to provide cardiovascular benefits in randomized clinical trials. For example, 1 g of n-3 fatty acids daily did not associate with conclusive effect on cardiovascular risk reduction in Outcome Reduction with an Initial Glargine Intervention (ORIGIN) trial [5]. However, in Reduction of Cardiovascular Events with Icosapent Ethyl-Intervention Trial (REDUCE-IT), patients who received 2 g of icosapent ethyl twice daily had 25% lower risk of ischemic events than placebo group [6]. Recently, genetic analysis indicated that the clinical benefit of lipid lowering therapy might attribute to the absolute change of ApoB-containing particles, which was mostly referred to cholesterol-rich LDL or TG-rich VLDL [24]. Thus, TG lowering drugs which could achieve specific reductions in TG-rich VLDL and its related parameters, might be beneficial [25]. In fact, in hypertriglyceridemic status, by activating CETP, the TGs from VLDL were transferred to LDL in exchange for cholesteryl esters, which led to increment in plasma LDL-TG levels [11]. Hence, finding the predictive value of LDL-TG in cardiovascular outcomes may provide new insight into the significance of TG lowering therapy.

In previous studies, controversial results were reported about the independent role of all TGs for predicting cardiovascular outcome. In Copenhagen Ischemic Heart Disease Study, increased concentrations of both calculated and measured RC were associated with increased all-cause mortality in patients with ischemic heart disease. However, according to Saeed et al. in ARIC study, RC and LDL-TG were associated with cardiovascular risk, but this association vanished for RC adjusted traditional risk factors including lipids. In a recent prospective study by Silbernagel et al. using the LURIC cohort (3140 participants and mean follow-up time 8.8 years), the fourth and fifth quantiles of LDL-TG had increased risk of cardiovascular mortality while no such association existed for the highest two quantiles of TG and VLDL-TG [26]. These studies indicated that LDL-TG may be the most powerful marker in predicting cardiovascular risk among all the TGs in lipoprotein subclasses. On the contrary, in the Atherothrombosis Intervention in Metabolic Syndrome with Low HDL/High Triglycerides and Impact on Global Health Outcomes (AIM-HIGH) trial, which examined the relationship of LDL-TG and cardiovascular events in a dyslipidemic population, LDL-TG was not associated with worse cardiovascular outcome [27]. However, on the same token,traditional lipid parameters,including LDL-C, HDL-C and non-HDL-C, were not predictive of clinical cardiovascular events either [28]. One of the possible reasons for these negative results was that niacin-induced compositional changes in lipoproteins might have affected the atherogenicity of the LDL-TG. Our real-world study, which was conducted in a large cohort of Chinese patients with stable CAD, not only validated the results from ARIC and LURIC studies but also demonstrated that the predictive value of LDL-TG for MACEs was superior to traditional lipid parameters including TG, LDL-C, non-HDL-C and ApoB.

The morbidity rate of DM and Pre-DM is increasing rapidly worldwide. The number of individuals with DM and Pre-DM may both reach 600 million in 2045 [13]. The coexistence of DM or Pre-DM and CAD is common. According to recent guidelines, patients with both ASCVD and DM are considered to be at a very high risk of cardiovascular events recurrence and mortality. Intensive LDL-C lowering treatments brought favorable cardiovascular outcome in those patients [29–31]. For example, achieving LDL-C < 1.4 mmol/L for those with combined status of DM and ASCVD was highly recommended by recent European guidelines on diabetes, pre-diabetes, and cardiovascular diseases [13]. However, even after intensive lowering of LDL-C, the absolute risk of events in DM patients is still higher than that in non-diabetic patients. Hypertriglyceridemia is the main feature of lipid disorder in DM and Pre-DM and could induce a sequence of compositional changes for lipid parameters including low HDL-cholesterol and type B pattern of LDL distribution [32, 33]. Despite the undisputable causal role of elevated LDL-C in patients with DM [34], TG-enriched lipoproteins were significantly elevated while plasma levels of LDL-C were usually within the normal range [12]. Considering the increased cardiovascular event rate in DM and pre-DM patients, identifying whether compositional features of diabetic dyslipidemia were impedimental factors for further risk reduction was crucial. Until now, the knowledge about LDL-TG in CAD risk prediction among DM and Pre-DM patients was very limited. In the present study, we investigated the prognosis of LDL-TG in real-world patients with different glucose metabolism status and stressed the predictive value of LDL-TG in DM and Pre-DM. In total population, DM but not Pre-DM patients presented higher risk of MACEs, which further validated the findings of previous studies [7, 26]. Notably, when LDL-TG level was incorporated as a stratifying factor in patients with different glucose metabolism status, the risk for those with high LDL-TG plus Pre-DM and high LDL-TG plus DM reached approximately 2-fold. Similarly, within DM group, patients with uncontrolled DM and high LDL-TG were least likely to be free of events, indicating an association of lack of glucose control with dyslipidemia. Our results may be instrumental in risk assessment for CAD patients with impaired glucose metabolism undergoing statin therapy, the so-called residual risk [35].

The results of this study have some practical implications. Firstly, measurement of LDL-TG may bring cardiovascular beneficial effect in clinical practice for its independent association with MACEs. Secondly, the results indicated that LDL-TG levels might be the most powerful marker among all the TGs in lipoprotein and a potential treatment target in DM. As previously reported, TNT trial, lipid lowering with statins reduced TGs in lipoprotein and resulted in a significant lower risk of MACEs [36]. Monoclonal antibodies to proprotein convertase subtilisin/kexin type 9 (PCSK9), alirocumab, could also significantly reduce plasma TG-rich lipoproteins in DM subjects [37]. More studies regarding the potential ways of LDL-TG reduction is in need. Thirdly, we once again concluded that the association between Pre-DM and MACEs might be attributable to the coexistence of other risk factors but our results also indicated that risk assessment by traditional lipid parameters may not be fully adequate [14].

This study has several limitations. First it only included Chinese patients with stable CAD, therefore, data from other ethnicity individuals is necessary; Second, the level of LDL-TG was only measured once in the fasting state and we cannot be sure about fluctuations of biomarkers during follow-up, even so the results were still robust; Third, although the incremental effect of adding LDL-TG into risk model was minor, it was consistent with previous findings. Finally, not all parameters reflecting glucose metabolism were measured due to the clinical and real-world nature of this study population.

## Conclusions

In conclusion, the current data showed, for the first time, that patients with high LDL-TG and DM or Pre-DM had significantly higher risk of MACEs, a finding that was in accord with robust epidemiological and genetic evidence of the active roles of TG-enriched lipoproteins in atherogenesis. It also suggested that incorporating LDL-TG for cardiovascular risk stratification might be beneficial in identifying higher risk patients with stable CAD and Pre-DM or DM.

## Supplementary information

**Additional file 1.** Additional figures and tables.

## Data Availability

The datasets used and/or analyzed during the current study are not publicly available but are available from the corresponding author on reasonable request.

## Abbreviations

NGR: Normal glucose regulation
Pre-DM: Pre-diabetes
DM: Diabetes mellitus
CAD: Coronary artery disease
MACEs: Major adverse cardiovascular events
PCI: Percutaneous coronary artery intervention
CABG: Coronary artery bypass grafting
BMI: Body mass index
HbA1c: haemoglobin A1c
TC: Total cholesterol
TG: Triglyceride
LDL-C: Low density lipoprotein cholesterol
HDL-C: High density lipoprotein cholesterol
LDL-TG: Low density lipoprotein triglyceride
GS: Gensini score
