# Supplementary material for: Long-term prognostic utility of low-density lipoprotein (LDL) triglyceride in real-world patients with coronary artery disease and diabetes or prediabetes
Source: Cardiovasc Diabetol. 2020 Sep 27;19:152. doi: 10.1186/s12933-020-01125-1 (PMC7520976; doi:10.1186/s12933-020-01125-1)
Supplement: Supplementary file 1 — Additional file 1. Additional figures and tables. [file 12933_2020_1125_MOESM1_ESM.docx]

**ADDITIONAL DATA**

**Additional Table S1 Univariate and multivariate linear regression analysis of the plasma LDL-TG levels with Gensini score**

| **Variables** | **Univariate linear regression** | | **Multivariate linear regression** | |
| --- | --- | --- | --- | --- |
|  | **Standardized coefficients** | **p-value** | **Standardized coefficients** | **p-value** |
| **TG** | -0.009 | 0.531 | 0.003 | 0.863 |
| **LDL-C** | 0.043 | **0.004** | 0.067 | **<0.001** |
| **HDL-C** | -0.059 | **<0.001** | -0.057 | **<0.001** |
| **Non-HDL-C** | 0.034 | **0.023** | 0.047 | **0.002** |
| **ApoB** | 0.049 | **0.001** | 0.065 | **<0.001** |
| **LDL-TG** | 0.040 | **0.007** | 0.048 | **0.002** |

Bold values indicate statistically significant.

Multivariate adjustments include age, sex, body mass index, smoking, hypertension, diabetes mellitus, family history of early coronary artery disease, and baseline statin use.

**ADDITIONAL DATA**

**Additional Table S2 Univariate and multivariate Cox proportional hazards regression analysis of the plasma LDL-TG levels with occurrence of MACEs**

| **Variables** | **Univariate Cox regression** | | **Multivariate Cox regression** | |
| --- | --- | --- | --- | --- |
|  | **HR (95%CI)** | **P** | **HR (95%CI)** | **P** |
| **Age** | 1.021(1.012-1.030) | **<0.001** | 1.018(1.008-1.028) | **<0.001** |
| **Male Sex** | 0.854 (0.709-1.030) | 0.099 | - |  |
| **BMI** | 1.003(0.975-1.032) | 0.821 | - | - |
| **LVEF** | 0.975(0.964-0.985) | **<0.001** | 0.979(0.968-0.989) | **<0.001** |
| **Hypertension** | 1.218(1.008-1.470) | **0.041** | - | - |
| **DM** | 1.305(1.096-1.554) | **0.003** | - | **-** |
| **Smoking** | 0.884(0.743-1.052) | 0.164 | - | - |
| **FH** | 0.848(0.652-1.105) | 0.222 | - | - |
| **TG (Per-SD)** | 1.087(1.002-1.180) | **0.046** | - | - |
| **HDL-C(per-SD)** | 0.976(0.893-1.067) | 0.599 | - | - |
| **LDL-C(per-SD)** | 1.116(1.027-1.212) | **0.009** | - | - |
| **GS** | 1.007(1.004-1.011) | **<0.001** | 1.006(1.002-1.009) | **0.002** |
| **LDL-TG (per-SD)** | 1.268(1.174-1.370) | **<0.001** | 1.328(1.202-1.467) | **<0.001** |
| **Baseline Statin use** | 0.772(0.638-0.933) | **0.008** | 0.774(0.636-0.941) | **0.010** |

Bold values indicate statistically significant.

BMI: body mass index; DM: diabetes mellitus; FH: family history of CAD; TG: triglyceride; LDL-C: LDL-cholesterol; HDL-C: HDL-cholesterol; LVEF: left ventricular ejection fraction; GS: Gensini score.

**ADDITIONAL DATA**

**Additional Table S3 Cox regression analysis according to different glucose metabolism**

| **Diabetic status** | **HR(95%CI)** | | |
| --- | --- | --- | --- |
| **(n, events/subjects)** | **Crude model** | **model 1** | **model 2** |
| **NGR (85/926)** | Ref | Ref | Ref |
| **Pre-DM (193/1789)** | 1.156(0.896-1.492) | 1.091(0.844-1.409) | 1.099(0.846-1.427) |
| **DM (229/1666)** | *1.529(1.192-1.957) | *1.417(1.102-1.822) | *1.328(1.025-1.721) |

* for p<0.05

NGR: normal glucose regulation; Pre-DM: pre-diabetes mellitus; DM: diabetes mellitus;

Model 1 adjusted for age and sex; model 2 adjusted for age, sex, body mass index, smoking, hypertension, family history of coronary artery disease, Gensini score, left ventricular ejection fraction, LDL-cholesterol, HDL-cholesterol, triglyceride, and baseline statin use.

**ADDITIONAL DATA**

**Additional Table S4 C-statistics of LDL-TG for predicting cardiovascular outcomes in pre-diabetes and diabetes groups**

| **Models** | **C-statistics (95%CI)** | **∆C-statistics (95%CI)** | **P** |
| --- | --- | --- | --- |
| **NGR Original Model** | 0.715(0.659-0.770) | Ref |  |
| **NGR Original Model +LDL-TG** | 0.715(0.660-0.771) | 0.001(-0.006-0.005) | 0.824 |
| **Pre-DM Original Model** | 0.687(0.648-0.727) | Ref | - |
| **Pre-DM Original Model +LDL-TG** | 0.704(0.664-0.742) | 0.016(0.004-0.033) | **0.028** |
| **DM Original Model** | 0.734(0.701-0.769) | Ref | - |
| **DM Original Model +LDL-TG** | 0.749(0.715-0.783) | 0.014(0.006-0.024) | **0.002** |

Bold values indicate statistically significant.

Variables include age, sex, body mass index, smoking, hypertension, family history of coronary artery disease, Gensini score, left ventricular ejection fraction, LDL-cholesterol, HDL-cholesterol, triglyceride, baseline statin use, and antidiabetic drugs (for DM only).

**ADDITIONAL DATA**

**Additional Figure**

**Additional Figure S1 Flowchart of the study**


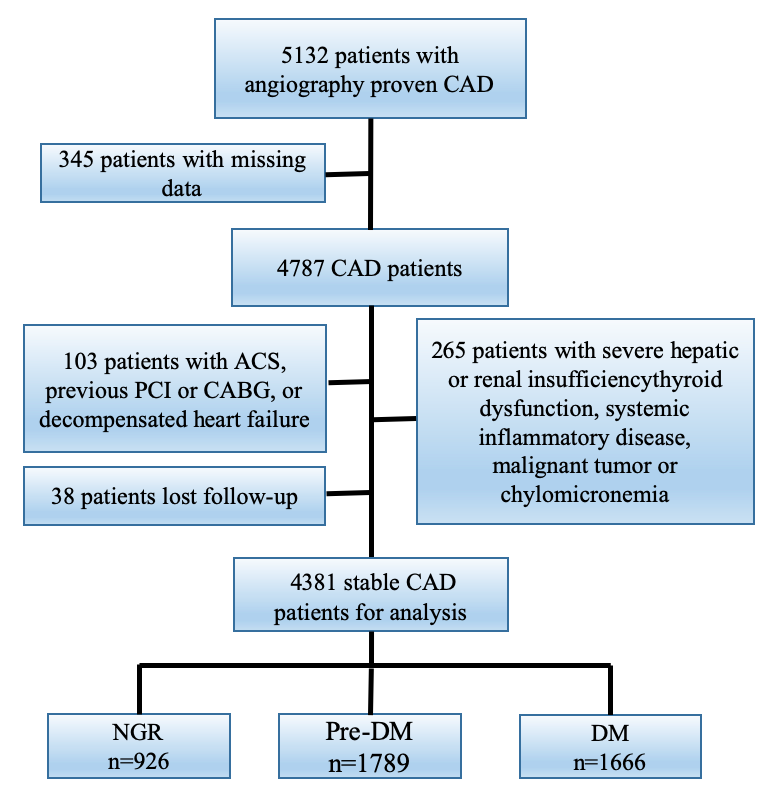


**ADDITIONAL DATA**

**Additional Figure**

**Additional Figure S2 coronary severity according to glucose metabolism status and LDL-TG levels**


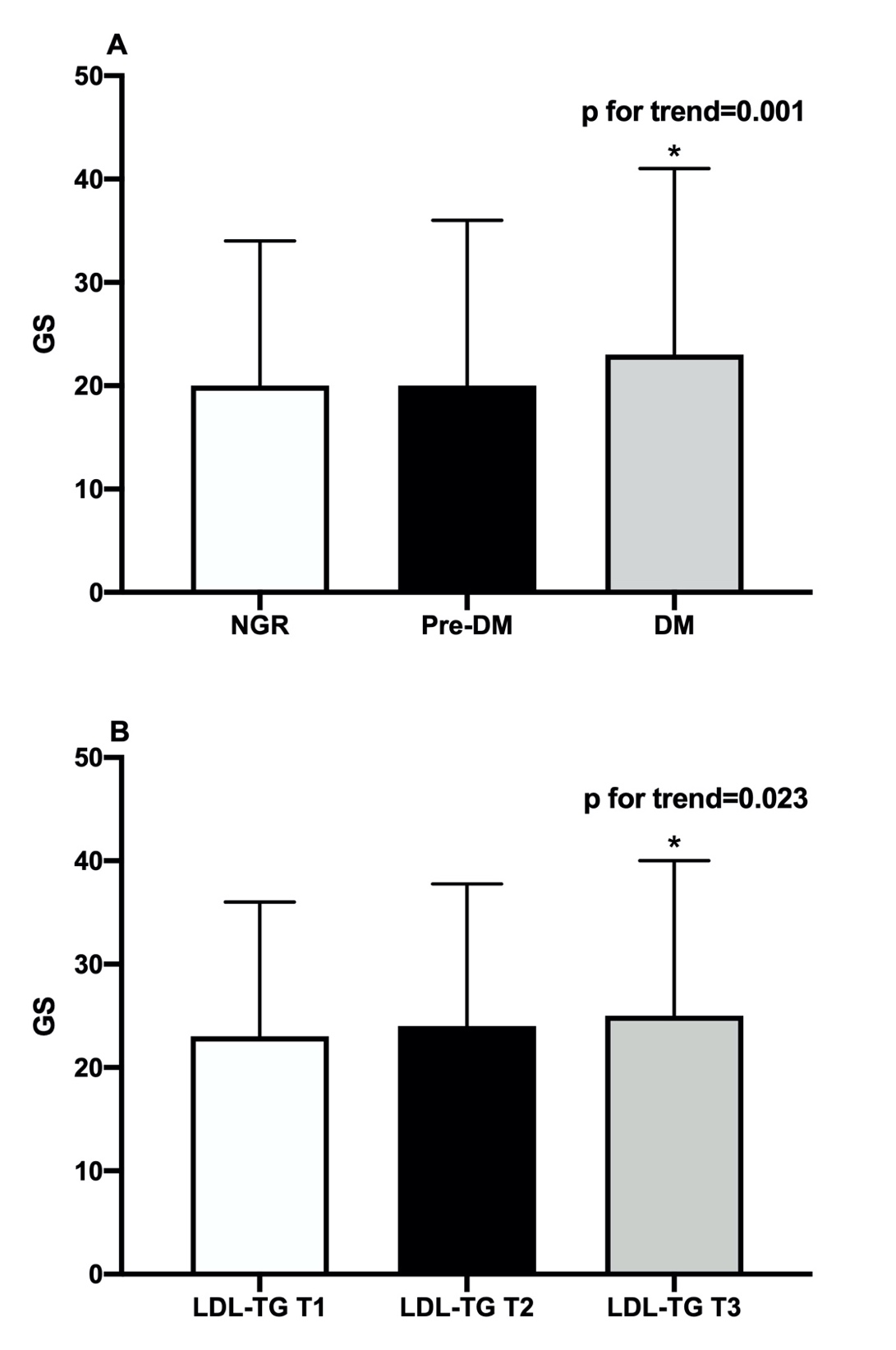
*****for p<0.05
